# Optimizing nutrition and metabolism in a severely burned patient during prolonged continuous renal replacement therapy: a case report

**DOI:** 10.3389/fnut.2025.1749501

**Published:** 2026-01-14

**Authors:** Haili Shan, Xiang Cheng, Yulu Hong, Tao Shen, Chuangang You, Chunmao Han

**Affiliations:** 1 Department of Pharmacy, The Second Affiliated Hospital, Zhejiang University School of Medicine, Hangzhou, China; 2 Department of Burns and Wound Care Center, The Second Affiliated Hospital, Zhejiang University School of Medicine, Hangzhou, China; 3 Department of Pharmacy, Haiyan People's Hospital, Jiaxing, China; 4 Department of Rehabilitation Department of Traditional Chinese Medicine, The Second Affiliated Hospital, Zhejiang University School of Medicine, Hangzhou, China

**Keywords:** burns, acute kidney injury, continuous renal replacement therapy, clinical nutritional, enteral nutrition, metabolism

## Abstract

It is rare for patients to survive and be discharged with extensive severe burns in combination with severe acute kidney injury (AKI). The case under discussion involved a patient who had sustained critical burn injuries, with 96% of the patient’s total body surface area affected, 80% of which were categorized as third-degree burns. Severe AKI was observed on the initial day following the burn injury, and continuous renal replacement therapy (CRRT) was commenced on the subsequent day. In response to inadequate oral intake, long-term enteral and supplemental parenteral nutrition was administered. To address the hypermetabolic state and promote wound healing, a dynamic, individualized nutritional support plan was implemented during the patient’s hospitalization. Regular monitoring of patients’ resting energy expenditure via indirect calorimetry ensures adequate energy and nutrient substrates are provided, while administering growth hormone to promote wound healing. Nutritional therapy efficacy is dynamically assessed through wound healing rate, clinical signs, serum prealbumin levels, and other relevant indicators to guide timely adjustments in nutritional intervention. On the 96th day, the patient’s wound had mostly healed and was transferred to a rehabilitation facility for continued treatment. This case presents an exceptionally complex clinical scenario, combining extensive burns with acute kidney injury. During a period of 95 consecutive days of CRRT, the burn wounds achieved substantial healing and the patient survived to discharge. A comparison with previously published case reports indicates that the case we report is distinguished by a larger total burn surface area, a prolonged course of CRRT, and a markedly more convoluted clinical trajectory. A precise, continuously refined nutritional support strategy and multidisciplinary team collaboration were instrumental in achieving a favorable outcome.

## 1 Introduction

Burns are defined as injuries to the skin and underlying tissues caused by heat, chemicals, electricity, or radiation, with thermal factors being the primary cause (1). The severity of burns is typically assessed comprehensively based on the total body surface area (TBSA) affected, the depth of the burn, along with the patient’s age. Burns affecting only the epidermis are classified as superficial first-degree burns; burns involving part of the dermis with redness and fluid exudation are second-degree burns; burns affecting the entire dermis are third-degree burns; and those penetrating to subcutaneous tissue, muscle, or bone are classified as fourth-degree burns. Clinically, minor burns are typically defined as those with TBSA< 10% and superficial characteristics. In contrast, severer burns encompass various scenarios, including >10% TBSA in elderly patients, >20% TBSA in adults, or >30% TBSA in children. Severe burns frequently entail a series of complications (2). Treatment for severe burns involves early fluid resuscitation, wound management, intensive care and organ function support, infection prevention and control, nutritional and metabolic support, as well as rehabilitation therapy (3). Nutritional interventions for burns present distinct challenges when compared to those for general critical illnesses. This is primarily due to the abnormal nutrient metabolism that is characteristic of burns (4, 5). The primary objectives of nutritional and metabolic therapy in burn patients are to deliver targeted nutritional support, stimulate protein synthesis, and accelerate wound healing (6–8).

The incidence of acute kidney injury (AKI) following burns ranges from 9 to 50%, with a mortality rate exceeding 80%. Continuous renal replacement therapy (CRRT) is the preferred supportive measure (9–11). CRRT clears toxins but also drives nutrient losses and raises energy expenditure, making nutritional management even more complex (12).

The recovery trajectory of patients with critical burns is rendered even more complicated when acute kidney injury (AKI) necessitates long-term continuous renal replacement therapy (CRRT), thereby significantly increasing the complexity of nutritional support.

## 2 Case presentation

On 22 September 2024, a 59-year-old male patient (height 170 cm, weight 77 kg) sustained full-body thermal fluid burns after coming into contact with molten iron water at temperatures between 700 and 800 °C at the workplace. Following initial cooling with cold water, the patient was urgently transported to a local medical center, where he underwent tracheotomy, central venous cauterization, resuscitation, and basic wound management. Approximately 5 h after the trauma, the patient was transferred to The Second Affiliated Hospital, Zhejiang University School of Medicine.

**Figure 1:**
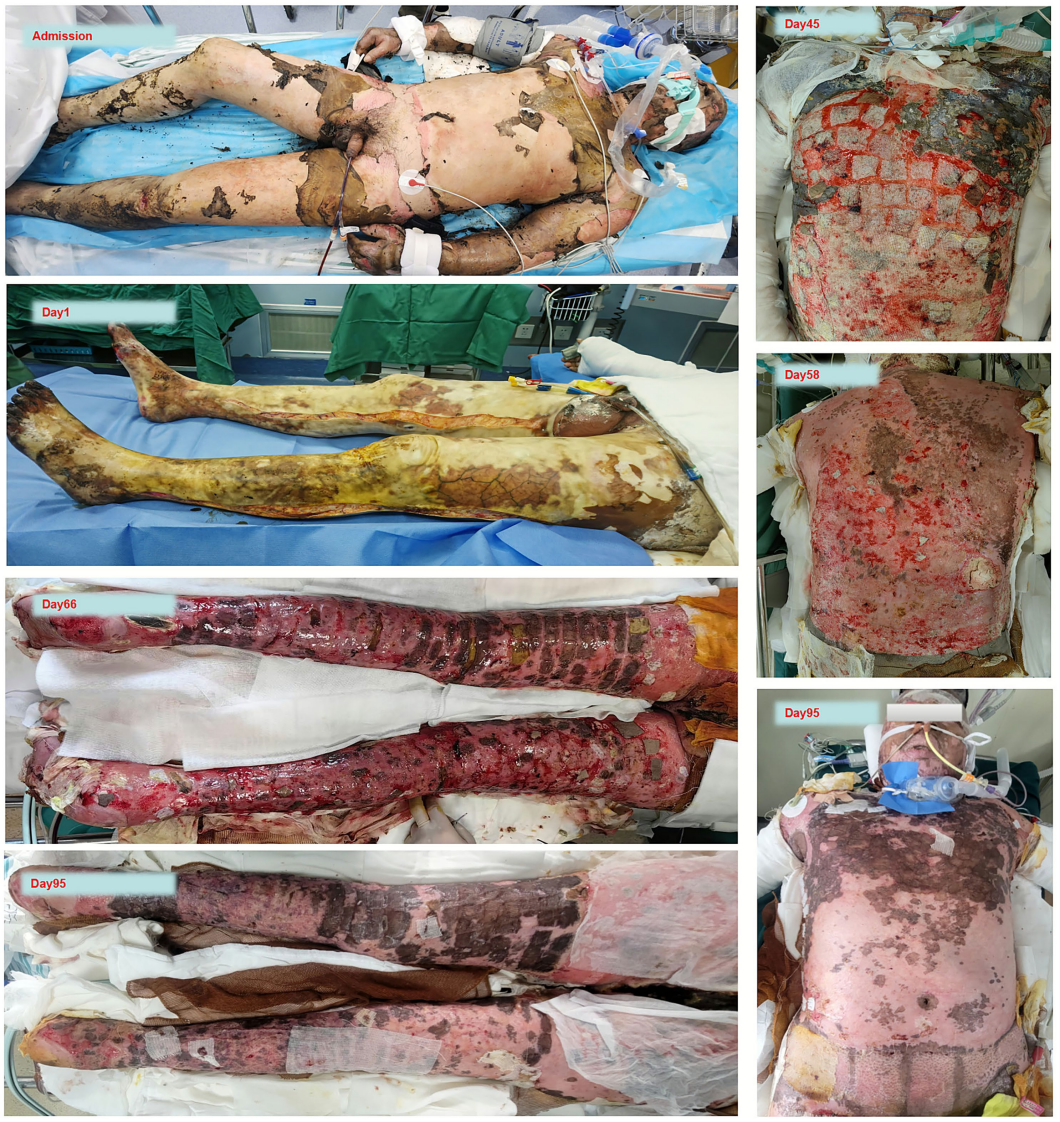
The patient’s burn wound from admission to discharge.

Admission findings included the following: temperature 35 °C, respiratory rate 8/min, heart rate 65/min, and blood pressure 101/78 mmHg. Physical examination revealed singed nasal hairs, hoarseness, and conjunctival oedema with ectropion. The burns were found to be distributed over a total of 96% of the body surface area, with 80% classified as third-degree (see Figure 1).

Laboratory tests indicated the following abnormal: Calcium: 1.85 mmol/L (↓); Hemoglobin: 195 g/L (↑); Albumin: 24.6 g/L (↓); D-dimer: 5180 μg/L FEU (↑); Creatine kinase: 324 U/L (↑); Aspartate aminotransferase: 132 U/L (↑); Total bilirubin: 32.0 μmol/L (↑); Creatinine: 112.5 μmol/L (↑); Arterial pH: 7.343 (↓); pCO₂: 47.0 mmHg (↑); Glucose: 14.50 mmol/L (↑); Lactate: 6.50 mmol/L (↑).

Early enteral nutrition is standard care for burn patients, as oral intake alone is insufficient to meet their heightened metabolic demands. Nasogastric feeding is the preferred route; however, post-pyloric feeding may be used as an alternative in cases of high aspiration risk. In the intensive care unit (ICU), EN is typically initiated with a fiber-supplemented polymeric formula (1, 13). Following emergency debridement surgery, the patient was transferred to the burn intensive care unit (BICU). In light of the potential for airway oedema resulting from inhalation injury, a nasogastric (NG) tube was inserted during the initial phase of fluid resuscitation, with the objective of establishing an enteral nutrition pathway. Approximately 8 h post-injury, enteral nutrition was initiated via the NG tube at an initial infusion rate of 20 ~ 30 mL/h. The Enteral Nutritional Suspension (Total protein fiber, TPF), with an energy density of 1.5 kcal/mL, was administered continuously through a feeding pump.

Adults with burns >20% TBSA require fluid resuscitation of 2 ~ 4 mL/kg/%TBSA within the first 24 h, provided intravenous access is available. Resuscitation should be titrated to a urine output of 0.5 ~ 1 mL/kg/h and a mean arterial pressure >70 mm Hg to prevent under- or over-resuscitation (14, 15). In order to address the shock that occurred during the initial phase of the burn injury, aggressive fluid resuscitation was initiated, with a total intake of 18,820 mL in the first 24 h. However, the patient’s mean urine output on Day 1 was only 16.8 mL/h, and the urine exhibited a tea-like appearance. During the second 24-h, the patient received a total fluid volume of 8,970 mL; however, urine output remained markedly low at 12.9 mL/h, and laboratory findings demonstrated progressive deterioration of renal function (blood urea nitrogen levels of 13.31 mmol/L and creatinine levels of 280.4 μmol/L). Following a comprehensive assessment, CRRT was initiated. Renal injury did not reverse during the patient’s period of hospitalization, and CRRT continued until discharge. Throughout the subsequent course of treatment, the patient’s fluid therapy regimen was continuously adjusted according to physiological requirements, cardiopulmonary status, and laboratory parameters. Nutritional support served as a substantial source of fluid intake and was integrated into the comprehensive fluid management strategy, along with other fluid sources such as intravenous infusions and oral rehydration.

After severe burns, patients transition from an initial hypometabolic phase to a prolonged hypermetabolic state. Given the inaccuracy of predictive equations, indirect calorimetry is the gold standard for assessing energy requirements (16), a metabolic cart was used to measure the patient’s energy requirements from day 3. According to the Indirect calorimetry (IC) data, the patient’s average resting energy expenditure (REE) during the first week was approximately 2,087 kcal/day. In consideration of the initial stress response and endogenous heat production following trauma, a nutritional support strategy was implemented that was below target energy requirements. From D 1 to D 3, the daily infusion volume was 500 mL (providing 750 kcal energy and 30 g protein); from D 4 to D7, this was gradually increased to 750 mL daily (providing 1,125 kcal energy and 45 g protein).

**Figure 2:**
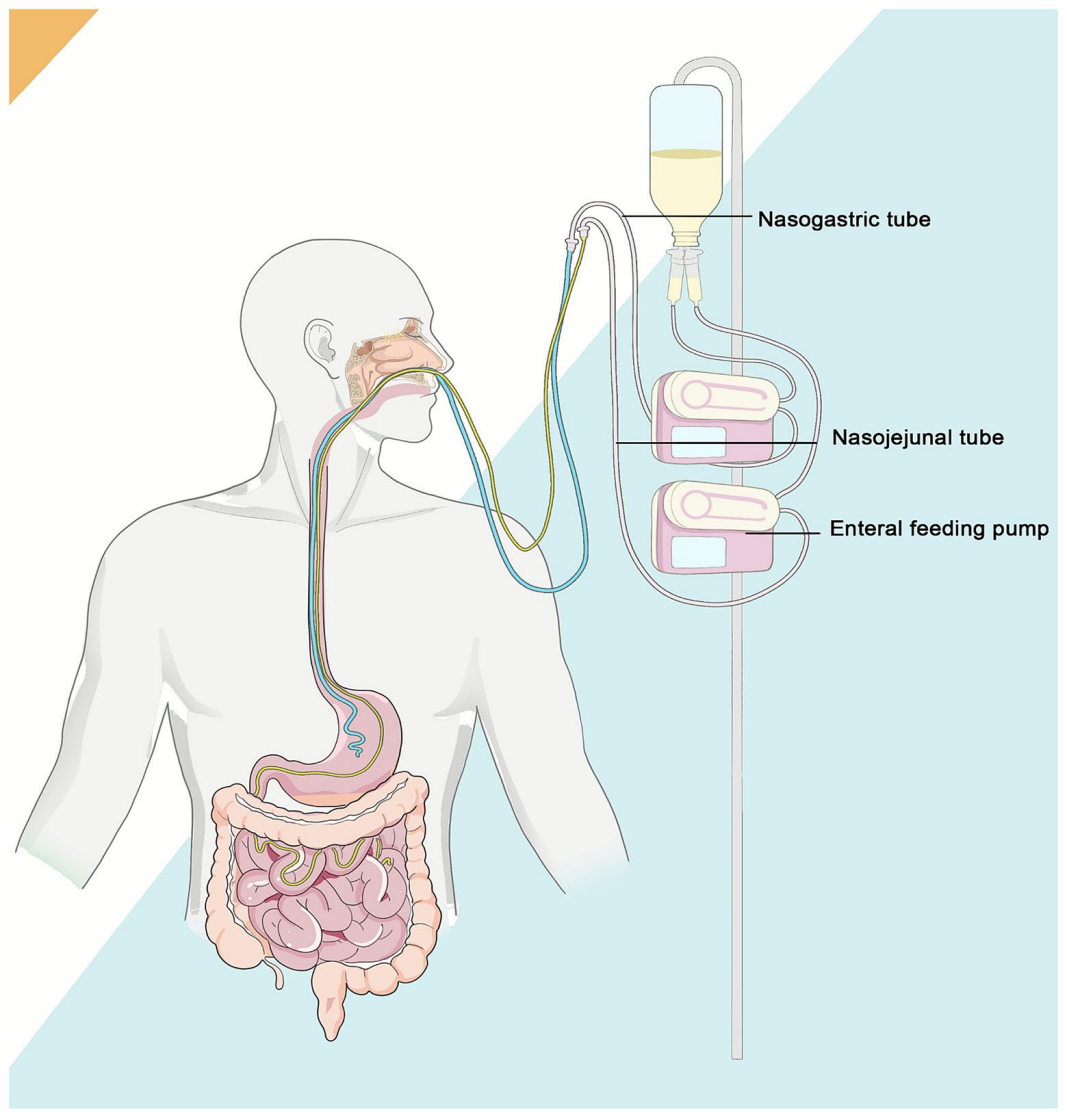
The schematic diagram of the dual-lumen (NG + NJ) enteral nutrition.

Considering that the patient was expected to undergo multiple surgeries and require long-term tube feeding, an additional Nasojejunal (NJ) tube was placed during the 2nd week of hospitalization. A dual-lumen (NG + NJ) enteral nutrition therapy was started to reduce the risk of gastrointestinal bleeding and aspiration pneumonia. The NG feeding delivered enteral nutrition at a low rate of 20 mL/h, while the NJ feeding provided a continuous infusion of nutritional solution at the standard rate of 50 ~ 70 mL/h. As shown in Figure 2, both tubes were connected to a single container of enteral nutrition and infused simultaneously at different rates. Feeding was well tolerated, with infusion volumes proportional to respective rates and adjusted temporarily based on clinical tolerance (e.g., gastric residual volume).

The patient’s REE increased to 2,700 kcal/day during the 2nd week, while serum prealbumin (PA) decreased from 107 mg/L to 87 mg/L. Considering the calories provided by intravenous fluids and CRRT dialysate (approximately 700 kcal/day), we increased the nutritional to 1,500 mL, providing 2,250 kcal of energy and 90 g of protein (calculated based on the product labeling). The patient’s energy intake approached the measured REE, with a protein intake of around 1.1 g/kg/day. To promote protein synthesis and wound healing, subcutaneous injections of recombinant human growth hormone 4 U/day were administered as an initial dose. Subsequently, the growth hormone dose was adjusted to 8 U/day in week 4 and further escalated to the target dose of 12 U/day by week 7, which was maintained until discharge. During the dose titration period, blood glucose monitoring frequency was increased to every 2 h, and glycemic fluctuations were promptly managed.

**Figure 3:**
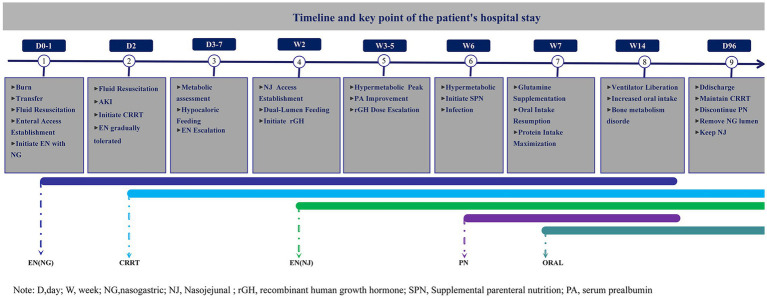
Timeline of nutritional treatment during the patient’s hospital stay.

From the 3rd week onwards, the patient entered a critical phase of wound repair and tissue regeneration. REE increased to 3,501 kcal/day (see Figure 3). From the 4th week onwards, the patient’s PA levels showed an upward trend, indicating improved nutritional status. By the 5th week, PA levels had risen to 93.5 mg/L, but then declined again from the 6th week (73 mg/L) and dropped to 42 mg/L by the 7th week. The patient experienced infection during this period, and we speculate that the decline in PA levels may be associated with the infection, although inadequate nutritional intake cannot be ruled out. As EN alone could not meet the patient’s high metabolic demands, supplemental Parenteral nutrition (PN) in the week 6, providing an additional 730 kcal of energy and 50 g of amino acids and 900 mL fluid volume per day (formulation provided in the Supplementary material). This increased the patient’s total energy intake to 2,808 kcal (35 kcal/kg/day) and their protein intake to around 122 g (1.5 g/kg/day).

From the 7th week onwards, an additional 10.8 g/day of glutamine (GLN) was added to the PN formula to promote wound healing and maintain intestinal mucosal barrier function. At this stage, the patient was able to swallow small volumes of liquid and oral medications. Therefore, an additional 30 g of GLN powder was administered orally each day, bringing the total protein intake to 224 g/day. At this point, the patient’s total daily energy intake reached 47 kcal/kg, with a protein intake of around 2.9 g/kg. Additionally, the growth hormone dose was adjusted to 12 U/day. This nutritional regimen was maintained until the patient was discharged.

At week 10 (day 68), the patient was weaned off mechanical ventilation and resumed spontaneous breathing. By week 14 (day 96), the area of the wound that was still unhealed had reduced to 5%, presenting as scattered, superficial lesions. The patient was transferred to a local medical institution for further treatment. At discharge, the patient’s vital signs were stable. Laboratory showed: WBC 16.8 × 10^9^/L (↑), PLT: 157 × 10^9^/L; Hb 49 g/dL (↓), ALT23 U/L, AST 37 U/L, Tbil 12.1 μmol/L, Total protein: 73.9 g/L, ALB 32.2 g/L (↓), PA 87 mg/L (↓).

During the hospitalization, the patient underwent 11 dermatological operations and sustained three septic-shock episodes, accepted 23.6 L plasma and 202 units of red-cell transfusion. The patient was still unable to obtain sufficient nutrition through oral intake at this time and the NJ feeding tube remained in place at discharge. As renal function had not yet recovered, the patient required ongoing CRRT therapy following hospital discharge. Notably, at the time of discharge, the patient exhibited symptoms of anemia and a disorder of bone metabolism (abnormal osteocalcin and β-collagen degradation products).

**Figure 4:**
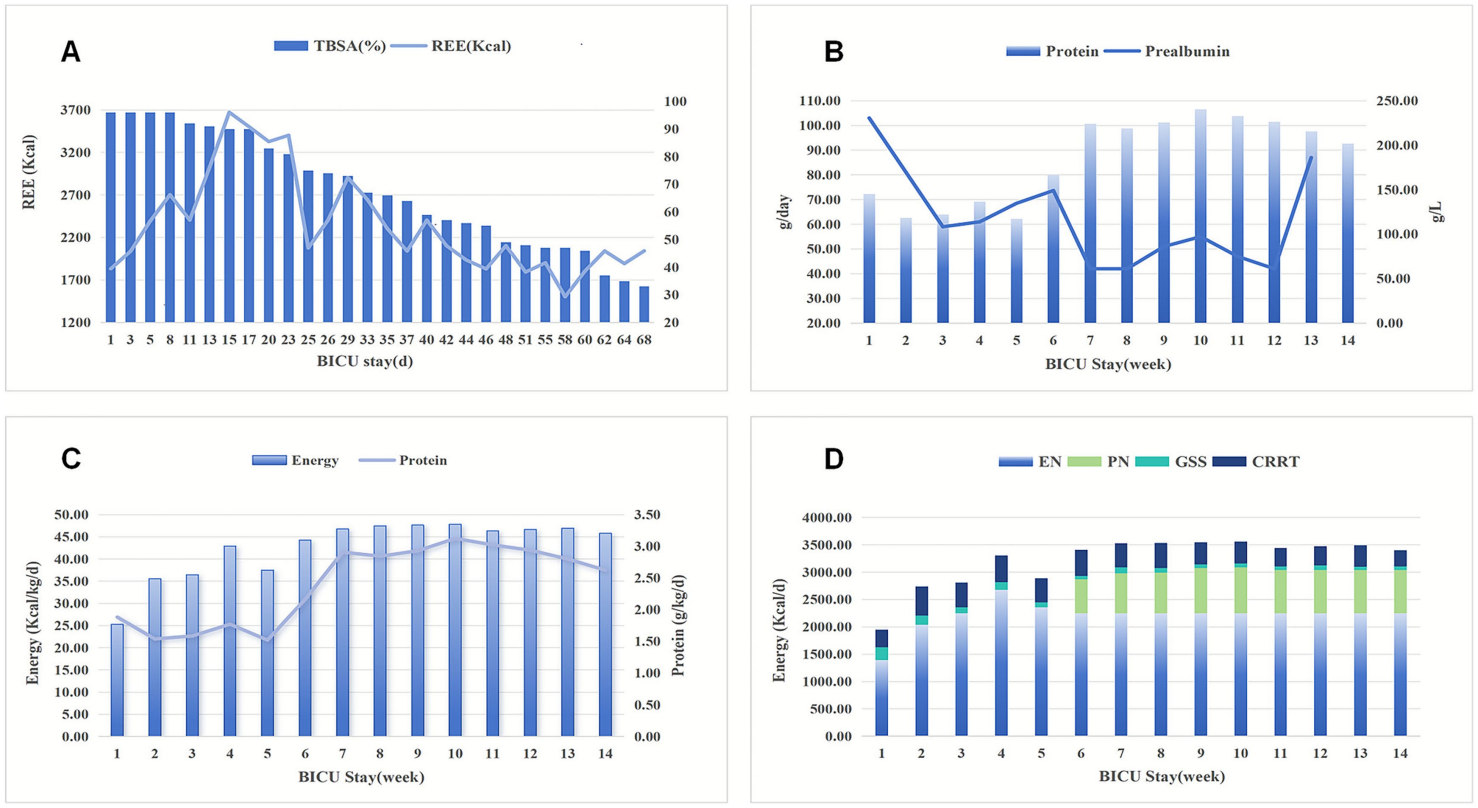
Nutritional parameters during the patient’s hospitalization. **(A)** REE and the remaining burn surface area (TBSA, %). The TBSA gradually decreased over time, while resting energy expenditure (REE, kcal/day) significantly increased in the early phase after burn injury and progressively declined as wound healing advanced; **(B)** The impact of protein intake on serum prealbumin levels during the BICU stay. PA levels increased concomitantly with higher energy and protein intake; however, these trends were interpreted as supportive indicators rather than definitive evidence of nutritional adequacy, given the influence of inflammation and infection; **(C)** Average daily energy and protein intake; **(D)** Energy and protein intake and sources during BICU stay; TBSA, total body surface area; PN, parenteral nutrition; EN, enteral nutrition; GSS, glucose-containing intravenous solutions; CRRT, citrate anticoagulant used in CRRT.

We have presented the patient’s nutritional plan during hospitalization in a visual manner through tables, images, and other means. Figure 1 depicts the patient’s burn wound. Figure 3 structurally illustrates the main nodes and strategies of nutritional therapy administered to the patient during their hospital stay. Figure 2 visually illustrates the schematic diagram of the dual-lumen enteral nutrition pathway. Table 1 summarizes the patient’s actual energy and protein intake during BICU hospitalization. Energy from enteral nutrition (EN), parenteral nutrition (PN), oral intake, human albumin, glucose solutions in intravenous fluids, and energy substrates (glucose and citrate) with CRRT were all included in total energy intake. The energy contribution of substrates was calculated as follows: 0.59 kcal/mmol for citrate, 4 kcal/g for glucose, 9 kcal/g for fat, and 4 kcal/g for protein (12, 17). Figure 4 systematically illustrates the dynamic changes in nutritional and metabolic status of a critically ill burn patient over a 14-week ICU hospitalization. It reflects multiple aspects, including energy and protein metabolism characteristics, adjustments in nutritional support strategies, and trends in key clinical indicators. Panel A shows the time-dependent reduction in total body surface area burned (TBSA, %). Meanwhile, resting energy expenditure (REE, kcal/day), assessed via indirect calorimetry (IC), was significantly elevated during the early phase of illness and remained at a high, fluctuating level in the initial weeks of hospitalization, indicative of a pronounced hypermetabolic stress response. Panel B demonstrates a positive correlation between protein intake and serum prealbumin levels; as protein intake increased, so did prealbumin concentrations, highlighting the impact of nutritional intake on hepatic synthetic function and short-term nutritional status. Panel C depicts variations in average daily energy intake (kcal/kg/day) and protein intake (g/kg/day) throughout the ICU stay, illustrating the progressive refinement of nutritional support protocols as the clinical condition evolved. Panel D, presented as a stacked bar chart, details the composition of daily total energy intake from various sources, including enteral nutrition (EN), parenteral nutrition (PN), glucose-containing solutions (GSS), and contributions related to continuous renal replacement therapy (CRRT).

**Table 1:** Daily average energy and protein intake of the patient during the BICU stay.

Week	Source of energy					*kcal/d*	*kcal/kg/d*	Source of protein				*g/d*	*g/kg/d*
	EN	ORAL	PN	GSS	CRRT			EN	PN	HSA	ORAL-GLN		
**1**	1392.86	0.00	0.00	235.35	320.08	1948.29	25.30	60.00	0.00	85.00	0.00	145.00	1.88
**2**	2035.71	0.00	0.00	174.64	526.51	2736.86	35.54	81.43	0.00	37.14	0.00	118.57	1.54
**3**	2250.00	0.00	0.00	110.50	447.98	2808.48	36.47	90.00	0.00	32.14	0.00	122.14	1.59
**4**	2678.57	0.00	0.00	141.00	486.39	3305.97	42.93	107.14	0.00	29.29	0.00	136.43	1.77
**5**	2361.43	0.00	0.00	89.71	435.80	2886.94	37.49	95.01	0.00	22.14	0.00	117.16	1.52
**6**	2250.00	0.00	623.31	59.16	475.71	3408.18	44.26	90.00	44.40	32.14	0.00	166.54	2.16
**7**	2250.00	120.00	727.20	112.03	441.59	3599.39	46.75	90.00	51.80	52.14	30.00	223.94	2.91
**8**	2250.00	120.00	744.57	79.97	461.01	3655.55	47.47	90.00	56.14	42.86	30.00	219.00	2.84
**9**	2250.00	120.00	826.57	67.66	402.86	3667.09	47.62	90.00	67.00	38.57	30.00	225.57	2.93
**10**	2250.00	120.00	839.43	72.15	400.27	3681.85	47.82	90.00	67.00	53.57	30.00	240.57	3.12
**11**	2250.00	120.00	788.00	69.58	337.98	3565.56	46.31	90.00	67.00	45.71	30.00	232.71	3.02
**12**	2250.00	120.00	788.00	87.23	347.35	3592.58	46.66	90.00	67.00	39.29	30.00	226.29	2.94
**13**	2250.00	120.00	788.00	60.86	393.20	3612.06	46.91	90.00	67.00	28.57	30.00	215.57	2.80
**14**	2250.00	120.00	788.00	69.19	298.14	3525.33	45.78	90.00	67.00	15.00	30.00	202.00	2.62

*PN, parenteral nutrition; EN, enteral nutrition, delivered via both nasogastric (NG; ~20 mL/h) and nasojejunal (NJ; 50 ~ 70 mL/h,), with the NJ route accounting for the majority of the volume and caloric intake; GSS, glucose-containing intravenous solutions; CRRT-AC, citrate anticoagulant used in CRRT; HSA, human albumin; ORAL-GLN, orally glutamine; REE, resting energy expenditure.

## 3 Discussion

In the early stages of severe burns, the patient’s metabolic rate can increase to more than twice the baseline level, and this hypermetabolic state often persists for two weeks or longer. This sustained hypermetabolic response has been shown to trigger severe catabolism, leading to immunosuppression and substantial depletion of lean body mass. Energy expenditure in patients suffering from burns fluctuates dynamically throughout the process of wound healing. While the REE predictive formulas often overestimate or underestimate actual energy expenditure. Malnutrition can impede wound healing, while overfeeding may lead to metabolic complications such as fatty liver, azotemia, and hyperglycemia (16). Indirect calorimetry (IC) is regarded as the gold standard for measuring REE, particularly in mechanically ventilated patients (18, 19). Therefore, guided by the established metabolic phases following burn injury, we employed a metabolic cart for IC to serially assess REE during the first 10 weeks of hospitalization, informing timely adjustments to the nutritional regimen.

The complexity of this patient’s clinical nutritional management stems primarily from the combined effects of severe burns, irreversible AKI, and dialysis therapy. While CRRT removes uremic toxins, it also results in the loss of small-molecule nutrients such as amino acids, glucose, and water-soluble vitamins (20). Research indicates that patients undergoing CRRT may lose approximately 17% of intravenously administered amino acids and 4% of glucose in the dialysate (21). Another important aspect to consider is the citrate anticoagulant used in CRRT, which partially enters the systemic circulation and undergoes subsequent metabolism, thereby providing additional energy (17). However, it is also important to note that continuous extracorporeal circulation results in some heat loss (22). Additionally, the use of CRRT may affect CO₂ clearance efficiency and interfere with the accuracy of REE measurements performed by the IC (23). During the early hypermetabolic phase of critically ill patients, the thermogenic effect of endogenous substrates is difficult to quantify precisely, further complicating metabolic assessment (24). This patient developed marked catabolism from severe burns and received prolonged CRRT, leading to significant ongoing losses of nutrients and energy. Standard nutritional protocols would likely have failed to meet actual requirements, resulting in a substantial supply–demand gap. Given this complex clinical condition, we routinely measured REE and used it-along with clinical status, evolving laboratory parameters, and energy contributions from concurrent therapies-to set higher targets for both caloric and protein intake. We also implemented enhanced monitoring of serum proteins, blood glucose, and lipid profiles to prevent undernutrition and overfeeding. This strategy was essential for achieving optimal wound healing.

A burn that affects more than 40% of the TBSA has the potential to affect the entire body. In patients with extensive burn injuries, an increase in metabolism is observed. This state of increased metabolism is known as hypermetabolism. A proportion of this energy is derived from the breakdown of the patient’s own muscles, resulting in muscle wasting. This deficit in energy and nutrient substrate can result in prolonged burn wound and donor site healing. The catabolic state can be treated with anabolic agents that reverse the protein breakdown. One of the anabolic agents recommended for such a treatment approach is recombinant growth hormone (25). Recombinant human growth hormone (rhGH) has been reported to attenuate catabolism and may contribute to improved wound healing outcomes in adults with severe burns, although its impact on hard clinical endpoints remains controversial. Moreover, rhGH’s impact on the patient’s blood glucose is an adverse effect that deserves clinical attention (26). In this case, rhGH was initiated at a low dose (4 U/day), consistent with expert consensus (7, 27) and manufacturer guidelines (0.2–0.4 IU/kg/day) (7) and titrated upward based on glycemic response, with intensified glucose monitoring to mitigate hyperglycemia and ensure a favorable efficacy–safety balance. We posit that, in the presence of adequate energy and nutritional substrates, growth hormone plays a pivotal role in promoting wound healing. However, this requires further validation through additional clinical studies in the future.

Glutamine (GLN) has been shown to have significant clinical importance for patients suffering from burns. A number of studies have previously demonstrated that levels of GLN decrease in patients undergoing major surgery, in intensive care units, and in burn patients. It has been demonstrated that a deficiency in GLN results in a loss of intestinal epithelial barrier function. The present study investigates the effects of GLN supplementation on intestinal mucosal atrophy during PN. GLN has been demonstrated to maintain levels of parenteral and intestinal immunoglobulin A, thereby significantly improving the outcomes of patients with burns by reducing mortality, shortening hospital stays and markedly decreasing Gram-negative bacteremia (28). ESPEN’s Guideline in Intensive Care Unit recommends that in patients with burns >20% body surface area, additional enteral doses of GLN (0.3 ~ 0.5 g/kg/d) should be administered as soon as EN is commenced (29). Consequently, for this patient, we administered supplemental GLN (0.5 g/kg/day) intravenously and orally for the majority of the hospitalization period.

Traditional nutritional monitoring indicators, such as body weight and muscle mass, have limited applicability for critically ill patients with extensive burns. However, there is currently no reliable biomarker to assess the response to nutritional therapy in critically ill patients (30). Serum PA is considered a sensitive predictor of clinical outcomes and a quality marker of nutritional support. Nevertheless, its susceptibility to inflammation limits its utility in critically ill patients (31). In this case, we interpreted a rising trend in PA levels as indicative of adequate nutritional support. As shown in Figure 4, PA levels increased concomitantly with higher energy and protein intake. Notably, the PA levels declined during episodes of infection, we did not attribute this decrease to insufficient nutritional intake. Throughout the treatment period, considering that CRRT may lead to energy and protein loss, particularly amino acids from parenteral nutrition (21), nutritional targets were set slightly above standard recommendations for typical burn patient (energy: 40 kcal/kg/day; protein: 2.5 g/kg/day) (32, 33). Under this regimen, the patient achieved satisfactory wound healing and did not develop overfeeding-related metabolic complications, such as hyperglycemia, azotemia, or fatty liver disease.

Critically ill patients are at high risk of stress-related mucosal disease (SRMD). SRMD-related gastrointestinal bleeding is also associated with an increased risk of death (34). Proton pump inhibitors and H2 receptor antagonists are currently the primary drugs used to prevent stress ulcers in critically ill patients (34–36). However, prolonged use of acid-suppressing drugs in intensive care settings has raised clinical concerns as it may increase the risk of adverse events, such as hospital-acquired pneumonia and *Clostridium difficile* infection (37, 38). Prior studies have suggested that enteral nutrition can reduce the incidence of gastrointestinal bleeding in both burn patients and critically ill patients who are on mechanical ventilation (39–41). Trophic feeding involves administering lower volumes of nutrients via the gastrointestinal tract. This can protect intestinal epithelial integrity, stimulate brush border enzyme secretion, enhance immune function and prevent bacterial translocation (42). NG and NJ feeding are the two most widely used methods of enteral nutrition. NG tube placement is simple and more closely mimics the natural feeding process. However, there is a higher risk of aspiration with this method in critically ill, comatose, or positional restricted patients. NJ feeding is used primarily for patients who cannot tolerate NG feeding. Conversely, their narrow lumen makes them susceptible to blockages and intestinal intolerance (43–45). NJ feeding bypasses the stomach, thereby reducing local irritation to the gastric mucosa and the neutralizing effect of gastric acid. Long-term use may increase the risk of stress-related mucosal diseases and gastrointestinal bleeding.

Although NG feeding is generally the preferred route for enteral nutrition in critically ill patients, the frequent surgical interventions and ongoing use of mechanical turning beds or medical fluidized beds in this patient necessitated NJ feeding as the primary enteral route to minimize the risks of tube displacement and aspiration. However, prolonged post-pyloric feeding can lead to gastric disuse, and we believe that trophic feeding may have beneficial effects. A study conducted in a neurosurgical intensive care unit (48) demonstrated that the combined utilization of NG and NJ feeding can serve as a management strategy for stress ulcers in critically ill patients. In this strategy, the primary function of the NG tube is to facilitate gastrointestinal decompression and drainage, while the NJ tube provides enteral nutrition. A similar dual-lumen enteral access was adopted in this patient, as illustrated in Figure 2. Two feeding tubes were performed in both nostrils, with the distal ends positioned in the stomach and jejunum, respectively. However, both tubes were connected to the same enteral nutrition container, thereby serving as shared enteral feeding pathways. The NG feeding delivered trophic feeding at a rate of 20 mL/h, while the NJ tube provided the primary nutritional feeding at 50 mL/h. It is the objective of this strategy to establish equilibrium between the feeding objective and the protection of the mucosa of the stomach. Despite the potential discomfort associated with dual-lumen placement, a comprehensive evaluation of risks and benefits has led to the conclusion that this approach is the optimal choice for the patient.

Research indicates that a multidisciplinary collaborative model in the nutritional management of critically ill patients contributes to improved clinical outcomes (46, 47). In the treatment of patients with severe burns, a multidisciplinary team comprising clinicians, nurses, pharmacists, nutritionists and rehabilitation therapists has been established. The formulation and adjustment of the overall treatment plan was led by physicians. The nutritionist managed complex nutritional issues and provided expert guidance, including aspiration risk assessment, initiation of PN, and glutamine supplementation, while nurses were responsible for executing medical orders and providing bedside care. Pharmacists were tasked with medication selection, dose optimization, and drug monitoring, and rehabilitation therapists conducted measurements of the patient’s REE and implemented limb function rehabilitation training. All members are responsible for conducting weekly summaries and discussions, as well as formulating the treatment plan for the following week. This collaborative model was found to play a crucial role in integrating the strengths of each specialty and achieving personalized treatment, thus serving as a key factor in the patient’s ultimately positive outcome.

This case study also presents certain limitations in treatment. Firstly, it is important to note that the measurement of weight during the patient’s period of hospitalization was challenging due to the use of thick gauze and dressings, complex treatment lines, and specialized beds (medical fluidized bed or turning beds). The energy and protein goals, which were calculated based on the subject’s pre-injury weight, may not align with their actual requirements. In estimating the energy contribution of nutritional substrates, we relied on the theoretical energy yield derived from the assumption of complete combustion-a standard approach in nutritional science. While this provides a useful idealized benchmark, it inevitably diverges to some extent from the actual metabolizable energy available to the patient. This discrepancy is a well-recognized limitation in the field and remains a technical challenge that current methodologies have yet to resolve. Secondly, the protective restrictions in place prevented regular assessment of muscle mass and quality, which limited the comprehensive evaluation of treatment outcomes. Furthermore, despite the patient’s PA levels demonstrating a consistent upward trend (see Figure 4), the final follow-up test could not be completed due to the patient and their family’s urgent request for discharge and rehabilitation. Furthermore, laboratory findings at the time of discharge indicated abnormal bone metabolism, potentially related to prolonged immobilization and calcium-phosphorus metabolism disorders induced by continuous CRRT. The absence of systematic bone density monitoring during the patient’s period of hospitalization represented a significant limitation in the management of the patient. These shortcomings provide important insights for the clinical management of similar cases.

## 4 Conclusion

In summary, in the nutritional management of long-term CRRT for severely burned patients with acute kidney injury, a multidisciplinary team comprising physicians, nurses, pharmacists, nutritionist, and traditional Chinese medicine rehabilitation therapists implemented precise nutritional monitoring and rehabilitation management. The patient’s individualized nutritional therapy plan was formulated based on a comprehensive consideration of multiple factors, including metabolic alterations induced by burns, indirect calorimetry measurements, the impact of CRRT on nutritional requirements, and the patient’s capacity for protein synthesis. Regarding enteral nutrition, a novel approach was adopted: a dual-lumen (NG + NJ) enteral feeding route was established, utilizing a TPF infusion to harness the synergistic effects of both conventional and trophic feeding strategies. In parallel, while closely monitoring metabolic parameters, subcutaneous growth hormone injections were administered to enhance anabolic processes and accelerate wound healing. A multifaceted approach was employed to assess the patient’s response to nutritional therapy. This included not only observing the patient’s mental status and rate of wound healing but also excluding inflammatory interference and using serum PA as a quantitative biomarker of protein synthesis. Additionally, rehabilitation therapists provided the patient with moderate physical exercise to preserve muscle strength. Ultimately, the patient’s deep burns were successfully healed. The present case offers a valuable opportunity for the acquisition of practical experience in a clinical context. Future clinical practice should emphasize enhanced monitoring of weight fluctuations, muscle mass/quality, and bone density to comprehensively evaluate nutritional status and treatment efficacy.

## Data Availability

The original contributions presented in the study are included in the article/Supplementary material, further inquiries can be directed to the corresponding author/s.
