# Supplementary figures and images for: Optimizing nutrition and metabolism in a severely burned patient during prolonged continuous renal replacement therapy: a case report
Source: Front Nutr. 2026 Jan 14;12:1749501. doi: 10.3389/fnut.2025.1749501 (PMC12849766; doi:10.3389/fnut.2025.1749501)

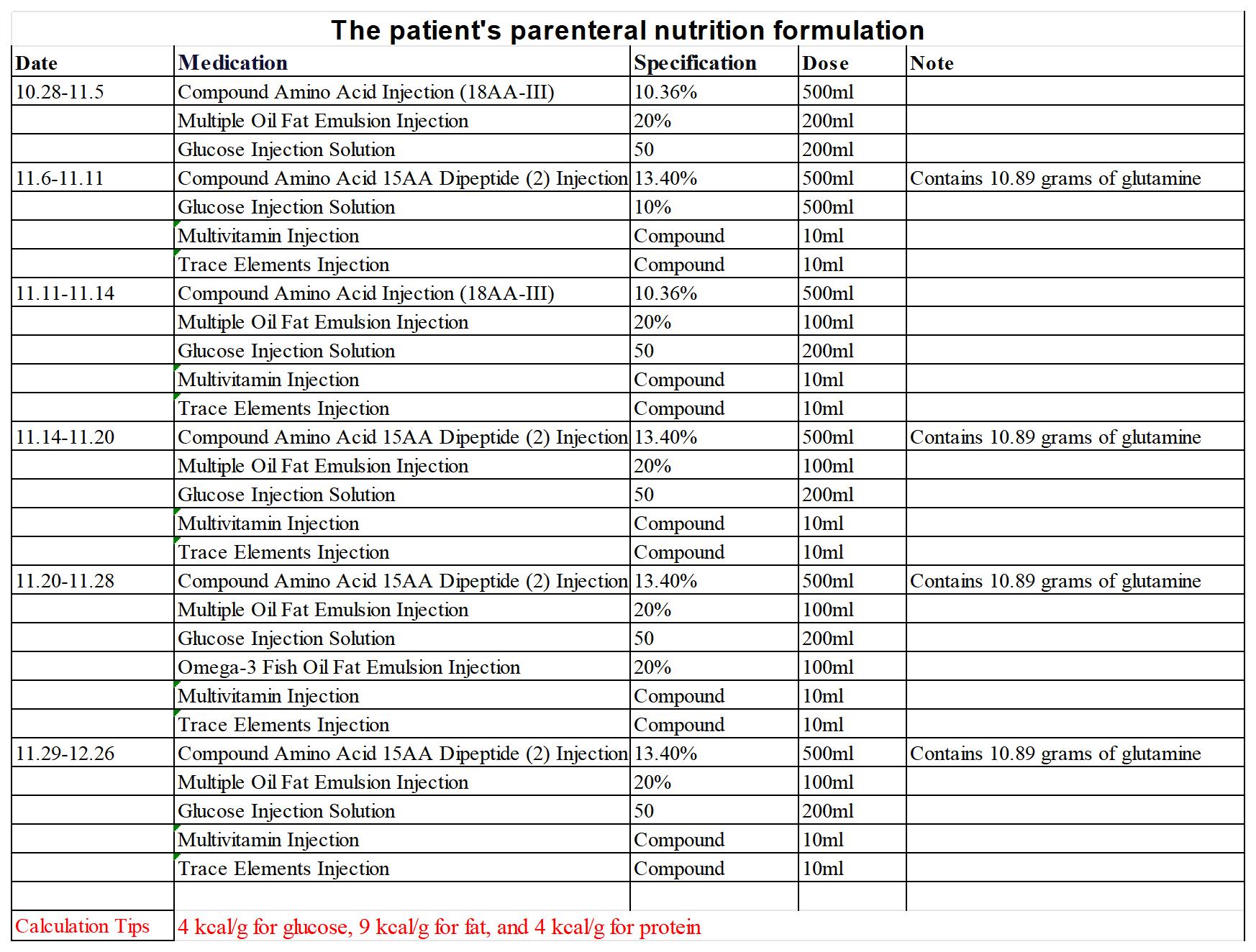

Supplement: Supplementary file 2 [file Image_1.JPEG]
